# Supplementary material for: Water acting as a catalyst for electron-driven molecular break-up of tetrahydrofuran
Source: Nat Commun. 2020 May 4;11:2194. doi: 10.1038/s41467-020-15958-7 (PMC7198510; doi:10.1038/s41467-020-15958-7)
Supplement: Supplementary file 1 — Supplementary Information [file 41467_2020_15958_MOESM1_ESM.pdf]

Supplementary Information

Water acting as a catalyst for electron-driven molecular break-up of tetrahydrofuran

Wang *et al.*

Supplementary Note 1. Intrinsic reaction coordinate (IRC) calculation

In the IRC calculations, first the equilibrium geometries were optimized with the second-order Møller-Plesset (MP2) method using the aug-cc-pVTZ basis set for THF, and THF·H_2O dimer and Becke's three-parameter hybrid functional combined with Lee-Yang-Parr correlation functional (B3LYP) with cc-pVDZ basis for THF·THF dimer. Based on the obtained geometry, the ground state potential energy surface (PES) of the single ionized molecule as a function of bond length was relaxed scanned, i.e. performing a geometry optimization at each scan step while keeping the scanned bond length as constant. All of the internuclear bond lengths of the ring were scanned in our calculation. Only initial C_β - C_β bond cleavage leads to the fragments observed in the experiment. Then the geometries corresponding to the maxima of the PES were extracted to perform the transition state (TS) optimization. This was calculated by B3LYP with the aug-cc-pVTZ basis set for THF^+, THF·H_2O^+ and with the cc-pVDZ basis set for THF·THF^+. The vibrational frequency, as well as the zero-point-energy, were obtained simultaneously with the configuration (equilibrium or TS geometry) optimization. For the equilibrium geometry, all of the frequencies are positive values while for the TSs one of them has an imaginary value. The geometry coordinates and vibration frequencies are summarized in the Supplementary Tables 1 - 10 where the imaginary frequencies are shown as negative values. The IRC calculation was performed under B3LYP/aug-cc-pVTZ level for THF^+ and THF·H_2O^+ and B3LYP/ cc-pVDZ level for THF·THF^+.

The IRC results of THF^+, THF·H_2O^+, and THF·THF^+ are shown in Supplementary Figures 1 - 3, respectively.

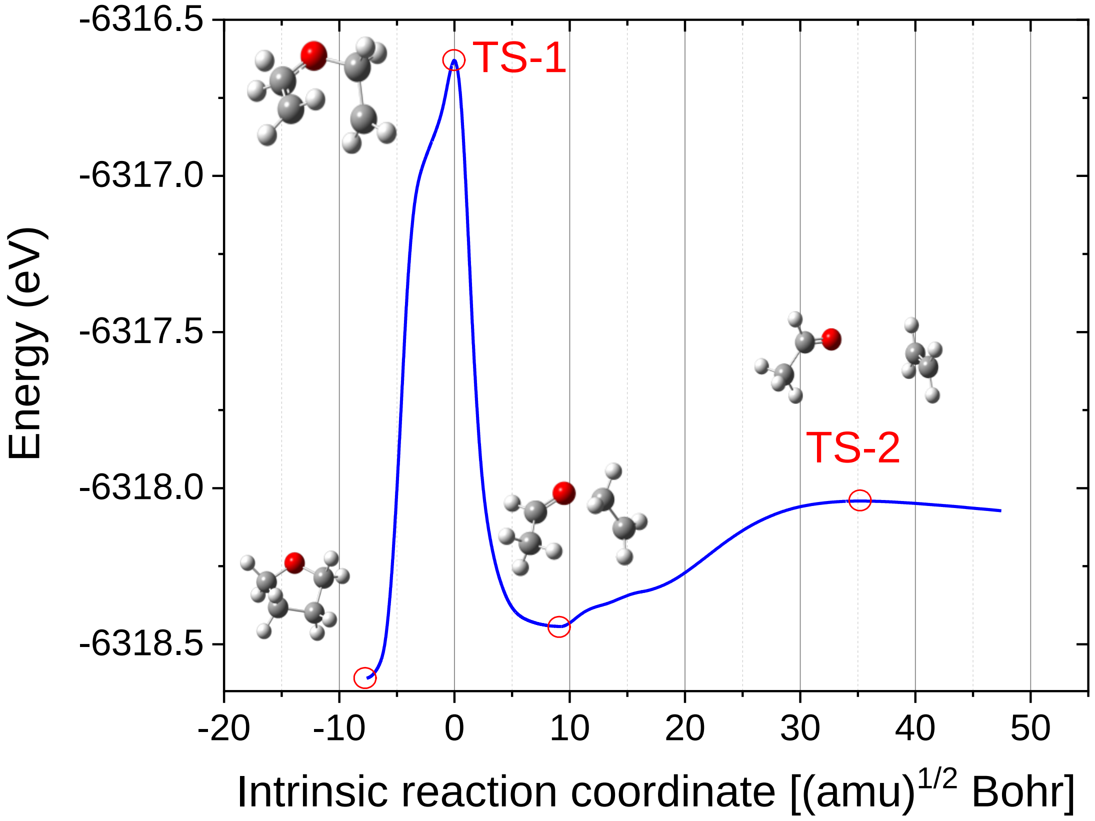

Supplementary Figure 1. Reaction pathway of the ring-break channel of THF^+. First, the intrinsic reaction coordinates were calculated for every transition state (TS) independently. Then these reaction pathways were combined by matching the geometries and energies of the individual pathways at connecting points. The calculations were performed using the B3LYP/aug-cc-pVTZ method.

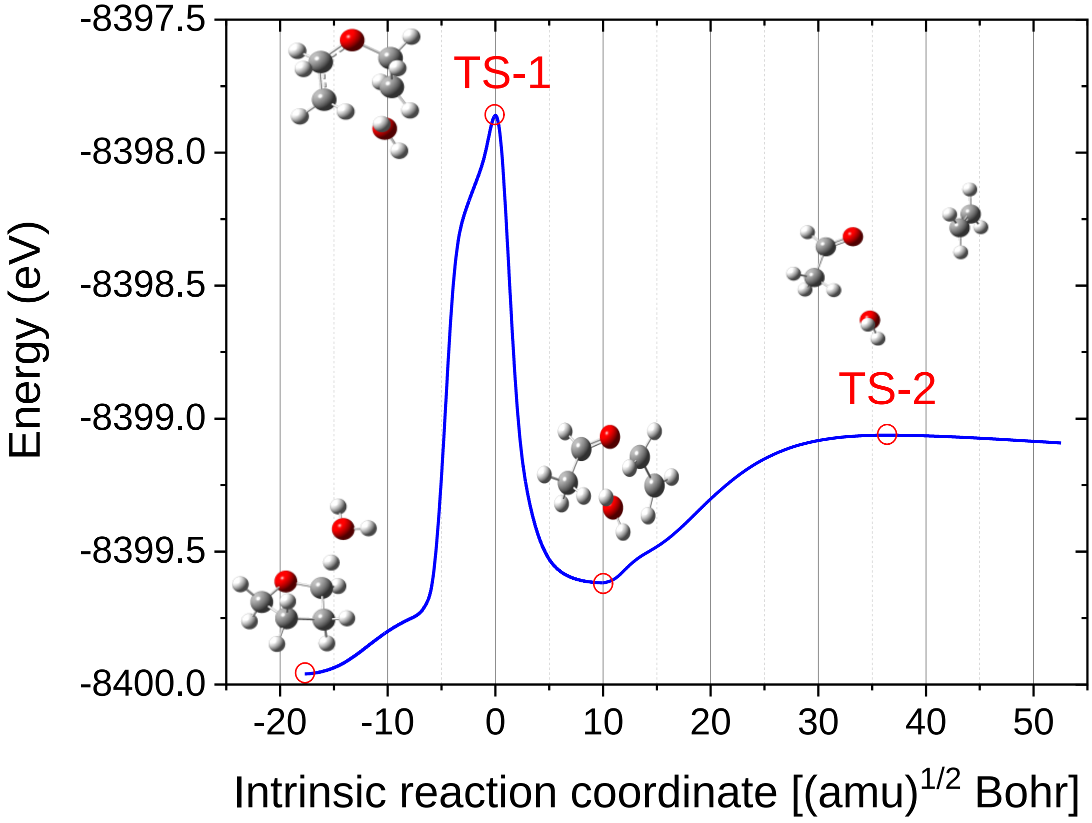

Supplementary Figure 2. Reaction pathway of the ring-break channel of THF·H_2O^+. As for Supplementary Figure 1.

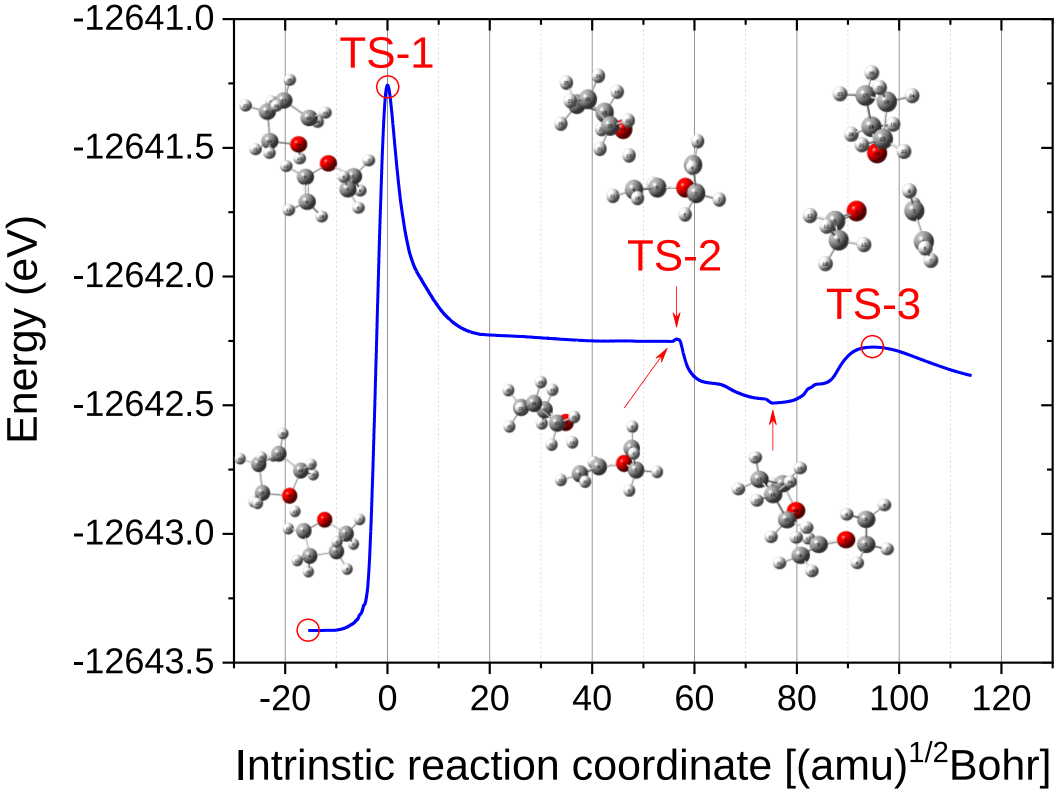

Supplementary Figure 3. Reaction pathway of the ring-opening channel of THF·THF $^{+}$. As for Supplementary Figure 1 except that the calculation was performed using the B3LYP/cc-pVDZ method.

Supplementary Note 2. Geometry of the neutral molecule and the transition state of the molecular cation and their vibrational frequencies.

Supplementary Table 1. Equilibrium geometry and vibrational frequencies of the neutral THF molecule obtained by MP2/aug-cc-pVTZ.

Geometry	Coordinates (Å)				Vibrational mode, Frequency(cm^{-1})			
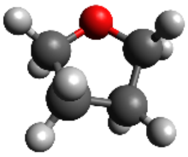	O	0.00000100	-1.24953300	-0.00000200	1	63.57	18	1319.62
	C	-1.16175600	-0.42013800	0.13945900	2	265.09	19	1346.33
	C	-0.72391100	0.98707300	-0.24325200	3	568.29	20	1371.65
	C	0.72390700	0.98707600	0.24325000	4	664.19	21	1401.40
MP2/aug-cc-pVTZ	C	1.16175800	-0.42013500	-0.13945600	5	852.59	22	1498.17
	H	-1.94728700	-0.81999700	-0.50053000	6	892.60	23	1507.68
	H	-1.50298500	-0.45335600	1.17836800	7	919.72	24	1538.90
	H	-0.75594800	1.11236400	-1.32622300	8	923.99	25	1548.84
	H	-1.33976700	1.75748600	0.21691800	9	949.97	26	3050.20
	H	1.33976200	1.75748900	-0.21692200	10	977.91	27	3053.67
	H	0.75594300	1.11237100	1.32622100	11	1050.87	28	3089.91
	H	1.50299400	-0.45335400	-1.17836200	12	1110.91	29	3092.27
	H	1.94728600	-0.81999100	0.50053900	13	1176.33	30	3130.23
					14	1191.83	31	3131.60
					15	1198.56	32	3156.57
					16	1271.95	33	3166.09
					17	1274.67		

Supplementary Table 2. Geometry and vibrational frequencies of TS-1 of THF^+ obtained by B3LYP/aug-cc-pVTZ.

Geometry	Coordinates (Å)				Vibrational mode, Frequency(cm^{-1})			
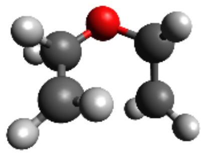	O	-0.72608900	0.10963900	1.89610300	1	-813.82	18	1228.50
	C	-0.81481500	-1.07774900	1.27317700	2	166.90	19	1249.52
	C	-0.31122300	-1.27701700	-0.03083900	3	252.35	20	1311.43
	C	-1.06330700	1.18281700	-0.24923000	4	265.67	21	1370.32
B3LYP/aug-cc-pVTZ	C	-0.36326900	1.23560700	1.05092100	5	396.83	22	1436.10
	H	0.14575700	-1.79934700	1.41383700	6	446.13	23	1467.25
	H	-1.63701100	-1.69474600	1.63116600	7	622.22	24	1481.56
	H	0.53736200	-0.72114200	-0.40202900	8	709.46	25	1523.88
	H	-0.60598400	-2.16687100	-0.57586800	9	754.95	26	2232.31
	H	-0.60562900	1.60588200	-1.13284700	10	830.64	27	3027.43
	H	-2.12178500	0.95949200	-0.28515400	11	905.74	28	3072.25
	H	0.72205600	1.29096300	0.94935600	12	948.55	29	3117.73
	H	-0.68605700	2.10937000	1.62560000	13	1007.39	30	3140.57
					14	1028.73	31	3146.26
					15	1066.60	32	3249.47
					16	1121.73	33	3257.40
					17	1209.48		

Supplementary Table 3. Geometry and vibrational frequencies of TS-2 of THF^{+} obtained by B3LYP/aug-cc-pVTZ.

Geometry	Coordinates (Å)				Vibrational mode, Frequency(cm^{-1})			
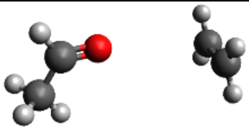	O	-0.90267600	-0.92564900	0.90698500	1	-29.62	18	1289.26
	C	-0.68429900	-2.10684300	0.96142100	2	7.85	19	1324.65
	C	0.17739800	-2.87513100	0.00700900	3	17.28	20	1353.17
B3LYP/aug-cc-pVTZ	C	-1.48555700	3.19910500	-0.70774900	4	43.51	21	1446.04
	C	-0.39462800	3.43298700	0.07870300	5	58.28	22	1449.16
	H	0.96521700	-3.36126900	0.58821400	6	62.14	23	1485.20
	H	-1.15633300	-2.68298000	1.78373000	7	166.13	24	1594.97
	H	0.59306300	-2.22855600	-0.75899000	8	473.27	25	1716.42
	H	-0.43576700	-3.66730500	-0.43034100	9	583.09	26	2878.85
	H	-1.40564500	3.18088500	-1.78641400	10	775.35	27	3029.54
	H	-2.46396600	3.03743900	-0.27572500	11	841.24	28	3088.11
	H	0.58151700	3.60695400	-0.35386600	12	879.92	29	3147.19
	H	-0.47678500	3.46348500	1.15683900	13	987.05	30	3152.37
					14	1060.94	31	3167.34
					15	1077.89	32	3235.44
					16	1098.89	33	3257.79
					17	1248.09		

Supplementary Table 4. Equilibrium geometry and vibrational frequencies of the neutral THF·H_2O dimer obtained by MP2/aug-cc-pVTZ.

Geometry	Coordinates (Å)				Vibrational mode, Frequency(cm^{-1})			
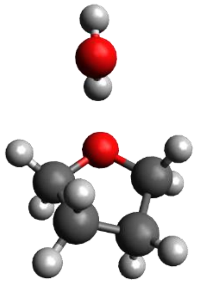	O	0.23690800	0.28060900	0.96191300	1	29.65	22	1272.04
	C	-0.30221400	-1.03330700	0.72474700	2	62.39	23	1281.25
	C	-0.95371600	-0.94731500	-0.64287600	3	98.13	24	1322.90
	C	-1.57091200	0.44933800	-0.57915500	4	137.13	25	1351.21
	C	-0.49748100	1.23928400	0.16570300	5	215.54	26	1375.45
	H	0.52353900	-1.73986200	0.77967100	6	279.14	27	1402.82
	H	-1.03625100	-1.26994200	1.50038700	7	393.59	28	1498.76
MP2/aug-cc-pVTZ	H	-0.18804700	-0.99391800	-1.41759300	8	570.69	29	1508.85
	H	-1.68533800	-1.73497100	-0.81267400	9	666.27	30	1534.22
	H	-1.78616600	0.87370900	-1.55752100	10	705.08	31	1546.29
	H	-2.49528800	0.42210400	-0.00154100	11	847.68	32	1648.05
	H	0.20716100	1.70695500	-0.52386300	12	892.22	33	3056.78
	H	-0.90758700	1.99912800	0.82965100	13	908.78	34	3080.29
	O	2.52569500	-0.01233700	-0.56116000	14	930.02	35	3093.75
	H	1.85011500	0.15618900	0.12118000	15	949.67	36	3097.85
	H	3.36298300	0.18642800	-0.13423200	16	980.01	37	3138.34
					17	1055.27	38	3151.53
					18	1091.27	39	3161.54
					19	1175.56	40	3171.48
					20	1193.81	41	3587.98
					21	1212.57	42	3903.78

Supplementary Table 5. Geometry and vibrational frequencies of TS-1 of $THF\cdot H_2O^+$ obtained by B3LYP/aug-cc-pVTZ.

Geometry	Coordinates (Å)				Vibrational mode, Frequency(cm^{-1})			
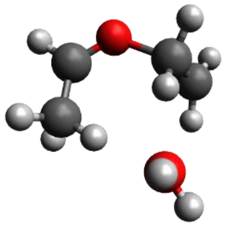	O	-0.72633100	0.10887900	1.90195800	1	-798.71	22	1125.32
	C	-0.81659100	-1.07580100	1.27196800	2	41.37	23	1218.98
	C	-0.30550500	-1.26530700	-0.03059300	3	70.92	24	1231.21
	C	-1.06757100	1.17602900	-0.24517600	4	119.18	25	1256.27
	C	-0.36451100	1.23539500	1.05293300	5	147.41	26	1318.47
	H	0.14230900	-1.79877000	1.40628800	6	169.14	27	1372.99
B3LYP/aug-cc-pVTZ	H	-1.64151400	-1.69098600	1.62612700	7	260.53	28	1439.81
	H	0.55804700	-0.71489200	-0.39710900	8	272.77	29	1464.98
	H	-0.61095100	-2.15375300	-0.57286300	9	310.02	30	1485.17
	H	-0.60170000	1.58338200	-1.13077300	10	314.91	31	1534.86
	H	-2.12800100	0.96219400	-0.27985900	11	392.11	32	1652.87
	H	0.71769300	1.28192800	0.93303700	12	451.07	33	2240.99
	H	-0.68537000	2.10860100	1.62825500	13	631.44	34	3033.51
	O	2.31059400	0.22820100	-0.96714400	14	737.13	35	3075.78
	H	3.17236300	0.15666600	-0.54112400	15	752.15	36	3091.97
	H	2.50890800	0.44409800	-1.88547800	16	832.05	37	3117.76
					17	911.00	38	3143.67
					18	965.68	39	3209.30
					19	1029.66	40	3254.48
					20	1043.74	41	3778.04
					21	1082.21	42	3863.31

Supplementary Table 6. Geometry and vibrational frequencies of TS-2 of THF·H_2O^+ obtained by B3LYP/aug-cc-pVTZ.

Geometry	Coordinates (Å)				Vibrational mode, Frequency(cm^{-1})			
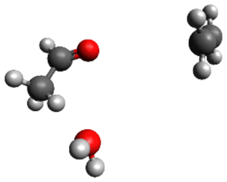	O	-1.04263600	-1.12668100	0.65195600	1	-23.36	22	1098.61
	C	-0.81132400	-2.30254400	0.73488400	2	8.08	23	1248.62
	C	0.41337600	-2.99332600	0.21860500	3	15.06	24	1273.93
	C	-1.66474800	3.62082100	-0.52164000	4	19.46	25	1329.88
	C	-0.38000200	3.37487900	-0.14738700	5	24.15	26	1353.64
B3LYP/aug-cc-pVTZ	H	0.87495600	-3.50741300	1.06631300	6	44.98	27	1451.95
	H	-1.56994000	-2.93877500	1.23825600	7	46.10	28	1465.05
	H	1.10134900	-2.29615500	-0.25502000	8	87.08	29	1485.37
	H	0.07725600	-3.76813000	-0.47621600	9	119.92	30	1601.57
	H	-1.96685600	3.57237100	-1.55909200	10	123.14	31	1640.71
	H	-2.42186400	3.88036600	0.20593400	11	173.53	32	1708.89
	H	0.37897100	3.12446600	-0.87600100	12	247.19	33	2862.25
	H	-0.07700000	3.43324200	0.88922500	13	258.38	34	3015.73
	O	2.66032500	-1.04153600	-1.26086400	14	470.58	35	3080.89
	H	3.52359300	-0.89263300	-0.86049100	15	637.51	36	3136.50
	H	2.83624300	-1.11808900	-2.20473000	16	778.30	37	3147.46
					17	841.01	38	3153.07
					18	878.17	39	3233.84
					19	985.41	40	3256.84
					20	1053.03	41	3783.90
					21	1073.19	42	3877.80

Supplementary Table 7. Equilibrium geometry and vibrational frequencies of the neutral THF·THF dimer obtained by B3LYP/cc-pVDZ.

Geometry	Coordinates (Å)				Vibrational mode, Frequency(cm^{-1})			
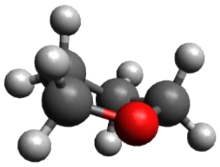	C	0.46951800	2.05943600	-1.07086300	1	33.15	37	1240.23
	O	-0.15425400	2.03014300	0.22407400	2	50.57	38	1241.10
	C	0.84974900	1.81813500	1.22207800	3	51.62	39	1253.22
	C	1.95955500	1.04212100	0.51493400	4	55.21	40	1254.67
	C	1.95955500	1.72564400	-0.86247800	5	62.52	41	1307.19
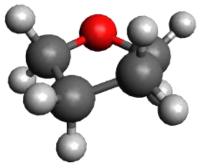	H	-0.03588200	1.31323100	-1.70958100	6	79.78	42	1307.26
	H	0.32195100	3.05305300	-1.53059400	7	97.97	43	1340.60
	H	1.22165800	2.79044000	1.60714800	8	100.26	44	1340.94
	H	0.38309400	1.27453800	2.05800700	9	273.42	45	1362.19
	H	2.92937600	1.10686000	1.03142800	10	280.40	46	1362.87
B3LYP/cc-pVDZ	H	1.67286400	-0.01813200	0.42451800	11	588.01	47	1386.39
	H	2.56136100	2.64918900	-0.83150000	12	588.55	48	1387.84
	H	2.36378700	1.08960700	-1.66404700	13	680.88	49	1463.03
	C	-0.84974900	-1.81813500	1.22207800	14	681.40	50	1464.04
	O	0.15425400	-2.03014300	0.22407400	15	829.44	51	1473.17
	C	-0.46951800	-2.05943600	-1.07086300	16	830.04	52	1477.66
	C	-1.95955500	-1.72564400	-0.86247800	17	885.75	53	1484.89
	C	-1.95955500	-1.04212100	0.51493400	18	886.04	54	1495.60
	H	-1.22165800	-2.79044000	1.60714800	19	911.74	55	1503.13
	H	-0.38309400	-1.27453800	2.05800700	20	912.59	56	1510.07
	H	0.03588200	-1.31323100	-1.70958100	21	916.05	57	2962.44
	H	-0.32195100	-3.05305300	-1.53059400	22	918.12	58	2963.46
	H	-2.36378700	-1.08960700	-1.66404700	23	930.72	59	3008.42
	H	-2.56136100	-2.64918900	-0.83150000	24	931.11	60	3009.28
	H	-1.67286400	0.01813200	0.42451800	25	968.74	61	3034.42
	H	-2.92937600	-1.10686000	1.03142800	26	969.94	62	3035.56
					27	1037.03	63	3048.53
					28	1037.80	64	3049.38
					29	1092.64	65	3051.10
					30	1097.56	66	3051.59
					31	1159.12	67	3080.36
					32	1159.13	68	3083.82
					33	1179.23	69	3098.02
					34	1179.90	70	3098.16
					35	1195.54	71	3111.56
					36	1195.84	72	3112.06

Supplementary Table 8. Geometry and vibrational frequencies of TS-1 of THF·THF⁺ obtained by B3LYP/cc-pVDZ.

Geometry	Coordinates (Å)				Vibrational mode, Frequency(cm^{-1})			
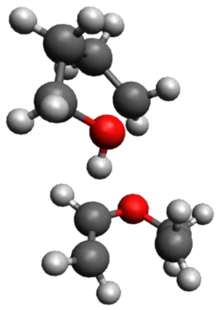	O	0.26767100	2.04310000	0.91147500	1	-648.24	37	1178.03
	C	1.53347500	1.91335100	1.65206100	2	41.75	38	1201.73
	C	2.44432500	0.94322300	0.96504300	3	47.20	39	1233.17
	C	1.74182700	1.91268000	-0.96109300	4	54.63	40	1235.77
	C	0.46995500	1.91611700	-0.42422500	5	79.14	41	1247.59
	H	1.98984100	2.91344100	1.72683200	6	104.00	42	1259.44
	H	1.22364700	1.59796700	2.66014900	7	150.45	43	1315.03
	H	3.51976200	1.13522400	0.98667800	8	223.10	44	1340.14
B3LYP/cc-pVDZ	H	2.16697800	-0.11656800	0.95350700	9	246.11	45	1351.55
	H	2.52775600	2.58354200	-0.61099700	10	273.29	46	1358.25
	H	1.86596100	1.58360700	-1.99801100	11	379.12	47	1377.50
	H	-0.45738500	1.79982400	-0.99509300	12	473.52	48	1413.87
	H	0.20170200	0.00914800	-0.46815100	13	558.91	49	1430.07
	O	-0.14170200	-0.92705500	-0.29304000	14	563.48	50	1446.44
	C	-0.68122200	-1.59420700	-1.53988700	15	602.63	51	1460.42
	C	-1.88961300	-2.35081900	-1.01835200	16	641.18	52	1466.15
	C	-2.44489900	-1.43386400	0.08720600	17	654.32	53	1481.13
	C	-1.19988600	-0.94432100	0.80497100	18	742.02	54	1484.33
	H	-0.80129700	-1.65173800	1.54234900	19	747.57	55	1498.82
	H	-1.22491100	0.07472700	1.20879400	20	766.30	56	1548.33
	H	-0.93571600	-0.78717300	-2.24128900	21	790.85	57	2942.08
	H	0.14235200	-2.20637000	-1.92407100	22	817.29	58	3056.28
	H	-2.61614100	-2.53136300	-1.82295600	23	839.71	59	3069.80
	H	-1.58515200	-3.32567900	-0.60734100	24	864.29	60	3076.17
	H	-3.00435500	-0.58950200	-0.34514100	25	885.50	61	3093.55
	H	-3.11297500	-1.96728700	0.77776700	26	904.15	62	3105.33
					27	908.09	63	3108.50
					28	923.11	64	3129.15
					29	949.75	65	3130.35
					30	976.04	66	3136.75
					31	983.93	67	3138.51
					32	1032.79	68	3163.05
					33	1048.68	69	3181.27
					34	1129.27	70	3186.58
					35	1131.43	71	3219.13
					36	1160.65	72	3232.98

Supplementary Table 9. Geometry and vibrational frequencies of TS-2 of THF·THF^+ obtained by B3LYP/cc-pVDZ.

Geometry	Coordinates (Å)				Vibrational mode, Frequency(cm^{-1})			
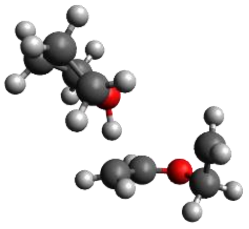	O	1.17329700	2.41562100	1.21730600	1	-534.12	37	1182.99
	C	2.51449600	1.88828000	0.93700800	2	29.29	38	1222.37
	C	2.59409100	0.42404200	1.14725800	3	40.24	39	1243.69
	C	0.19368400	1.54591500	-0.82977700	4	54.50	40	1252.16
	C	0.20863100	2.26520200	0.35174700	5	68.24	41	1259.97
	H	2.81510000	2.19762900	-0.07743800	6	113.40	42	1282.58
B3LYP/cc-pVDZ	H	3.13875900	2.44512900	1.65847400	7	122.22	43	1313.83
	H	3.37828000	-0.14377600	0.64190500	8	183.44	44	1351.59
	H	2.07497900	-0.02754200	1.99636000	9	187.74	45	1355.77
	H	1.13373500	1.23074000	-1.29319200	10	259.25	46	1363.61
	H	-0.62329100	1.79295000	-1.51620600	11	285.51	47	1389.66
	H	-0.70306200	2.76064400	0.70981800	12	321.39	48	1394.70
	H	-0.28568100	0.23870700	-0.32681500	13	433.49	49	1422.77
	O	-0.64958400	-0.84072200	-0.04215600	14	471.39	50	1431.15
	C	-0.12652300	-1.90839600	-0.93735700	15	564.73	51	1459.94
	C	-1.27818500	-2.89783700	-1.03547600	16	569.52	52	1462.61
	C	-2.51907800	-1.99191900	-0.96952700	17	604.00	53	1468.17
	C	-2.12977900	-0.96027500	0.08031200	18	692.54	54	1481.37
	H	-2.31889900	-1.29188100	1.11021100	19	718.07	55	1487.20
	H	-2.54981700	0.04325000	-0.07197400	20	802.21	56	1500.77
	H	0.11642300	-1.43854500	-1.90277700	21	822.50	57	1658.63
	H	0.78357300	-2.29402900	-0.46240700	22	836.14	58	3025.92
	H	-1.22566800	-3.48609000	-1.96215800	23	850.27	59	3065.44
	H	-1.25796600	-3.59670600	-0.18475800	24	882.87	60	3070.61
	H	-2.70136300	-1.50822600	-1.94226600	25	915.16	61	3074.24
	H	-3.42998300	-2.53520900	-0.68276500	26	928.65	62	3084.04
					27	949.48	63	3087.01
					28	974.81	64	3120.30
					29	995.57	65	3127.64
					30	1017.87	66	3133.02
					31	1046.43	67	3146.79
					32	1048.40	68	3152.01
					33	1055.64	69	3158.46
					34	1124.81	70	3160.49
					35	1144.40	71	3217.67
					36	1168.83	72	3266.10

Supplementary Table 10. Geometry and vibrational frequencies of TS-3 of THF·THF⁺ obtained by B3LYP/cc-pVDZ.

Geometry	Coordinates (Å)				Vibrational mode, Frequency(cm^{-1})			
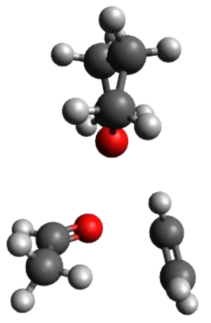	O	0.07205800	1.80939800	1.13765800	1	-42.71	37	1184.66
	C	3.07026700	0.87303600	1.02257200	2	14.15	38	1226.80
	C	1.91270900	0.26091200	1.42811500	3	30.60	39	1232.92
	C	0.52966300	2.71956100	-1.06832300	4	41.17	40	1242.23
	C	-0.24168800	2.50571900	0.18922800	5	46.43	41	1299.41
	H	3.42057000	0.80342200	-0.01129500	6	64.45	42	1303.33
	H	3.69215400	1.43485100	1.72534200	7	83.23	43	1329.18
	H	1.33525300	-0.38251700	0.75594000	8	88.68	44	1337.98
B3LYP/cc-pVDZ	H	1.58766000	0.32520800	2.46818600	9	95.01	45	1340.18
	H	0.71729100	3.80110200	-1.18571500	10	139.03	46	1352.12
	H	-0.10556900	2.42010000	-1.91981900	11	152.87	47	1358.28
	H	-1.21515300	3.04891000	0.26771300	12	174.94	48	1420.38
	H	1.47120100	2.15804800	-1.06843500	13	191.97	49	1422.79
	O	-0.54413600	-0.94084200	-0.38606200	14	250.31	50	1455.94
	C	-0.55854800	-1.81083100	-1.54164000	15	296.40	51	1456.39
	C	-1.43189800	-3.00781600	-1.17018300	16	517.55	52	1457.95
	C	-2.42140500	-2.38187200	-0.17509300	17	577.95	53	1460.17
	C	-1.52934600	-1.39144100	0.56889800	18	671.07	54	1481.98
	H	-0.99585400	-1.87528900	1.41121000	19	778.48	55	1575.91
	H	-2.04313000	-0.50154000	0.96712400	20	819.43	56	1734.69
	H	-0.98202400	-1.23470300	-2.38708700	21	829.33	57	2931.61
	H	0.48489000	-2.06059400	-1.79332000	22	850.88	58	2982.21
	H	-1.91909100	-3.45741300	-2.04628700	23	865.52	59	2995.20
	H	-0.82687900	-3.78710700	-0.67891500	24	877.61	60	3037.19
	H	-3.22584600	-1.84842100	-0.70733500	25	901.72	61	3053.98
	H	-2.88562000	-3.11492700	0.49885700	26	904.95	62	3058.73
					27	932.29	63	3073.83
					28	951.99	64	3074.71
					29	980.37	65	3102.52
					30	1034.28	66	3123.76
					31	1036.77	67	3129.68
					32	1041.12	68	3133.26
					33	1101.32	69	3146.96
					34	1115.07	70	3179.03
					35	1123.56	71	3242.13
					36	1166.73	72	3264.77

Supplementary Note 3. Energy levels

In this work, the total energy of the molecular system includes electronic energy, vibrational energy and thermal free energy,

$$E_{total} = E_e + \sum_i (n_i + \frac{1}{2})\hbar\omega_i \ + G,$$

where E_e is the electronic energy, n_i is the vibrational quantum number with a frequency of ω_i. G is the Gibbs free energy which includes two parts,

$$G = H - TS,$$

in which H is the enthalpy, S is the entropy and T is the temperature.

For a supersonic cold-molecule target, the excitation of initial vibrational states is negligible. Therefore, only the zero-point vibration ($n_i = 0$) is considered for the initial state. For vertical ionization from the HOMO, the excited vibrational state of the cation cannot be determined precisely. Therefore, in our calculation only the zero-point-energy is considered for the vibrational energy part.

The potential barrier from the vertical ionization point to the highest TS is

$$E_a = \Delta E_e + \Delta E_{\mathrm{zero}} + \Delta G,$$

where, $\Delta E_e = E_e^{TS} - E_e^{+}$, $\Delta E_{\mathrm{zero}} = \sum_j \frac{1}{2}\hbar\omega_j^{TS} - \sum_i \frac{1}{2}\hbar\omega_i^{+}$, $\Delta G = (H^{TS} - TS^{TS}) - (H^{+} - TS^{+})$, are the energy differences between electronic parts, zero-point-energy, and Gibbs free energy, respectively.

In the article, we show the energy levels including the electronic energies with zero-point-energy corrections. The following figure (Supplementary Figure 4) presents the PES of the ring-break channel of THF^+ for comparison with that of $THF{\cdot}THF^+$ shown in the article in Fig. 4c for the same calculation level. The geometries were optimized using B3LYP/cc-pVDZ. The electronic energies were determined using CCSD(T)/cc-pVDZ. The zero-point-energy corrections were obtained by B3LYP/cc-pVDZ.

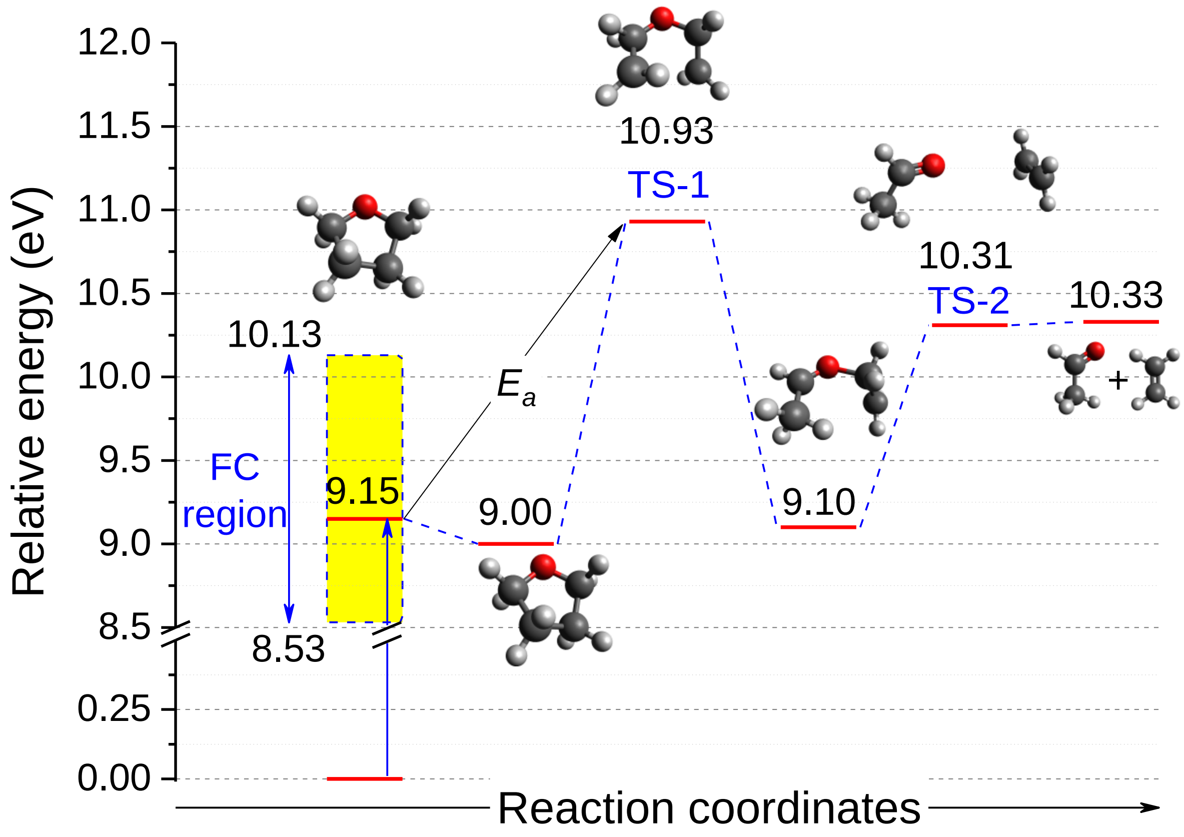

Supplementary Figure 4. PES of the THF$^+$ ring-break channel for the same calculation level as used in the PES calculation for THF·THF$^+$ shown in the article in Fig. 4c. The large activation energy (E_a = 1.8 eV) from the vertical ionization point to the TS-1 state inhibits this reaction channel. FC refers to the Franck-Condon region which is marked by the yellow bar. All energies include zero-point vibrational energy corrections. The calculation was performed using the B3LYP/cc-pVDZ method.

Supplementary Note 4. Franck-Condon region

The vibrational motion of the nuclei influences the ionization energy. The electronic energy difference between the cation and neutral state, ΔE_e, is not a single line but a broad distribution, i.e. the Franck-Condon (FC) region, which corresponds to the overlap between initial and final vibrational levels. Here we estimate the FC region profile as follows,

(i) Calculate the normal modes and vibrational frequencies of the molecule (MP2/aug-cc-pVTZ for THF and THF·H_2O, B3LYP/cc-pVDZ for THF·THF);

(ii) Perform thermal sampling for the neutral molecule from quantum harmonic oscillator distribution (5000 samples using the Newton-X package [1-2]);

(iii) Calculate the HOMO ionization potentials of the sampled geometries using the outer-valence Green's function method (OVGF).

In the second step, only the zero-point vibration is considered. In the third step, we choose the basis set to ensure that the ionization potential of the equilibrium geometry matches previous results obtained by the CCSD(T) method (deviation less than 0.1 eV). Here, the cc-pVDZ basis set is selected for THF and THF·H_2O and 6-31G* basis set is selected for THF·THF, respectively.

Supplementary Note 5. Activation energy as a function of hydration number

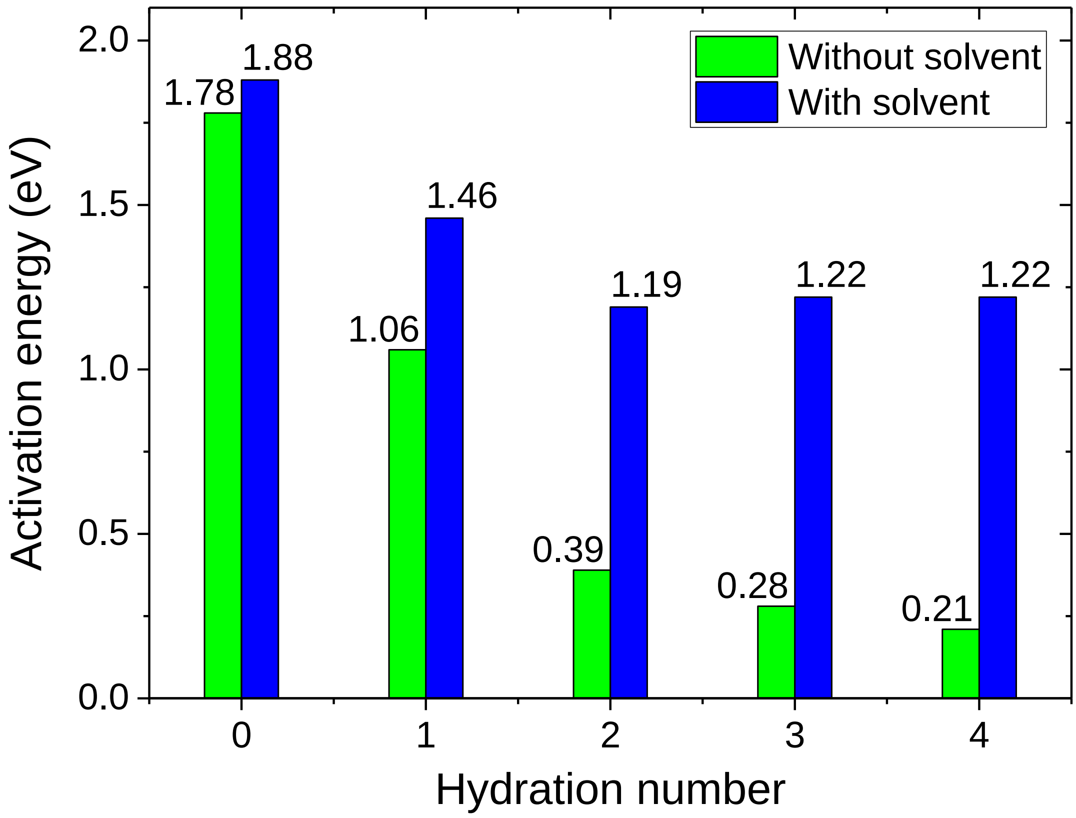

Supplementary Figure 5. Activation energy as a function of hydration number. 0 corresponds to the THF monomer. For each of the hydrated THF molecules, the equilibrium geometry of the neutral state and the transition state corresponding to the ring-opening and proton transfer are optimized with and without solvent effect using the polarizable continuum model (PCM). All of the calculations are performed at B3LYP/cc-pVDZ level.

The geometry and vibrational frequencies of TS of each of the hydrated THF are as given in the Supplementary Tables 11-20.

Supplementary Table 11. Geometry and vibrational frequencies of the transition state of THF^{+} without solvent effect obtained by B3LYP/cc-pVDZ.

Geometry	Coordinates (Å)				Vibrational mode, Frequency(cm^{-1})			
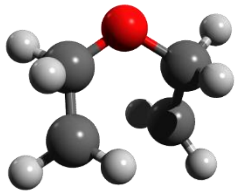	O	-0.72934300	0.11368100	1.90613500	1	-805.50	18	1216.62
	C	-0.81591900	-1.07358400	1.28031200	2	163.49	19	1235.67
	C	-0.31580100	-1.27063900	-0.03529000	3	254.91	20	1303.60
	C	-1.05902700	1.17334000	-0.25296500	4	266.22	21	1349.40
	C	-0.36258900	1.23477600	1.05636100	5	401.10	22	1412.19
B3LYP/cc-pVDZ	H	0.15260000	-1.80559500	1.40595400	6	445.72	23	1440.10
	H	-1.63985800	-1.70298300	1.64631600	7	621.40	24	1452.43
	H	0.55051300	-0.72003700	-0.40837800	8	701.84	25	1515.68
	H	-0.62107300	-2.16658800	-0.58879200	9	746.21	26	2228.00
	H	-0.59674800	1.60472700	-1.14540100	10	832.68	27	3032.53
	H	-2.13083800	0.95236200	-0.28787300	11	899.92	28	3081.55
	H	0.73444600	1.29735300	0.95991900	12	937.65	29	3126.42
	H	-0.69635900	2.12008500	1.62789500	13	990.11	30	3137.18
					14	1028.85	31	3144.28
					15	1051.68	32	3256.36
					16	1111.71	33	3264.49
					17	1196.77		

Supplementary Table 12. Geometry and vibrational frequencies of the transition state of THF^{+} with the solvent effect obtained by B3LYP/cc-pVDZ.

Geometry	Coordinates (Å)				Vibrational mode, Frequency(cm^{-1})			
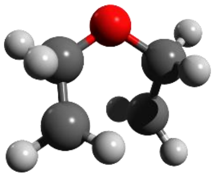	O	-0.71733300	0.12001700	1.92941100	1	-794.94	18	1192.70
	C	-0.81884700	-1.06990600	1.27720900	2	174.02	19	1236.45
	C	-0.31510900	-1.23651500	-0.02946300	3	248.09	20	1292.50
	C	-1.05906800	1.14368600	-0.24477000	4	274.22	21	1346.35
	C	-0.36051700	1.23375300	1.05964500	5	393.09	22	1397.50
B3LYP/cc-pVDZ	H	0.14015900	-1.80927400	1.38116800	6	454.11	23	1421.49
with solvent effect	H	-1.65179100	-1.69421600	1.62297200	7	624.15	24	1432.68
	H	0.55593300	-0.68330500	-0.38552400	8	706.77	25	1503.36
	H	-0.62968100	-2.12056700	-0.59356800	9	729.51	26	2281.78
	H	-0.58618700	1.54529200	-1.14381500	10	829.95	27	3047.69
	H	-2.12896900	0.91628300	-0.26997500	11	884.04	28	3102.04
	H	0.73357800	1.28480800	0.94382300	12	944.89	29	3146.78
	H	-0.69216200	2.12684400	1.61708100	13	1005.32	30	3158.81
					14	1031.37	31	3160.92
					15	1050.74	32	3270.77
					16	1113.34	33	3280.68
					17	1180.85		

Supplementary Table 13. Geometry and vibrational frequencies of the transition state of THF·H_2O^+ without solvent effect obtained by B3LYP/cc-pVDZ.

Geometry	Coordinates (Å)				Vibrational mode, Frequency(cm^{-1})			
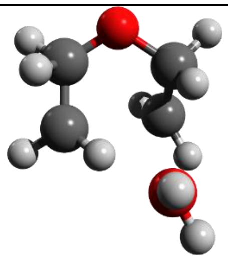	O	-0.65638700	0.13123300	1.88672100	1	-787.81	22	1117.06
	C	-0.78501700	-1.06127300	1.27638800	2	59.44	23	1212.34
	C	-0.32209000	-1.27449200	-0.05086400	3	111.57	24	1219.87
	C	-1.06455300	1.15057400	-0.27503100	4	159.66	25	1250.82
	C	-0.30658600	1.23252900	0.99860500	5	177.47	26	1315.62
	H	0.17350200	-1.80889400	1.37319700	6	180.18	27	1357.77
B3LYP/cc-pVDZ	H	-1.60865500	-1.66976600	1.67634800	7	270.84	28	1415.78
	H	0.56083200	-0.74555000	-0.44784100	8	292.43	29	1440.28
	H	-0.67254500	-2.17143600	-0.57601600	9	296.96	30	1464.05
	H	-0.62881300	1.54522800	-1.19607000	10	325.36	31	1526.59
	H	-2.14093000	0.95080300	-0.26110700	11	393.47	32	1661.46
	H	0.77986500	1.25490600	0.82180300	12	455.04	33	2234.91
	H	-0.59607900	2.13272800	1.56923100	13	635.20	34	3033.16
	O	2.21395400	0.25460100	-0.84493800	14	736.04	35	3042.33
	H	3.08144400	0.04313200	-0.46357300	15	753.53	36	3107.43
	H	2.43392600	0.62154000	-1.71640800	16	836.94	37	3125.06
					17	908.38	38	3140.52
					18	965.86	39	3207.30
					19	1029.55	40	3260.94
					20	1051.24	41	3745.97
					21	1080.12	42	3832.99

Supplementary Table 14. Geometry and vibrational frequencies of the transition state of $THF\cdot H_2O^+$ with the solvent effect obtained by B3LYP/cc-pVDZ.

Geometry	Coordinates (Å)				Vibrational mode, Frequency(cm^{-1})			
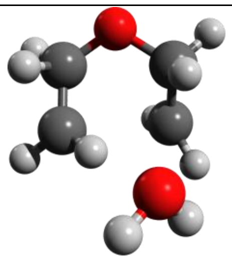	O	-0.77229800	0.15562400	1.90599200	1	-777.69	22	1121.36
	C	-0.82575600	-1.05078900	1.27517300	2	85.43	23	1188.94
	C	-0.21185600	-1.25066100	0.02109000	3	99.31	24	1200.47
	C	-0.93426600	1.11801500	-0.31667400	4	123.77	25	1243.74
	C	-0.34594100	1.24828200	1.03789200	5	137.37	26	1305.51
	H	0.11613900	-1.78902300	1.47894400	6	192.02	27	1352.06
B3LYP/cc-pVDZ	H	-1.68963600	-1.66109300	1.56592200	7	243.19	28	1403.37
with solvent effect	H	0.70005900	-0.71006000	-0.27494400	8	263.81	29	1427.26
	H	-0.49349400	-2.14918900	-0.53867800	9	298.89	30	1438.05
	H	-0.38434000	1.48267900	-1.18715400	10	330.02	31	1518.09
	H	-2.00024000	0.89603600	-0.42479400	11	398.41	32	1613.80
	H	0.75285100	1.29397700	1.00394700	12	460.73	33	2302.67
	H	-0.72344200	2.15442300	1.54216600	13	638.65	34	3050.36
	O	2.43101600	0.36721400	-0.71031900	14	723.77	35	3060.38
	H	2.87307500	-0.35755700	-1.18323300	15	770.28	36	3115.56
	H	1.96999500	0.83798500	-1.42488500	16	835.48	37	3145.99
					17	892.97	38	3157.64
					18	965.05	39	3229.98
					19	1033.78	40	3269.84
					20	1056.68	41	3728.84
					21	1097.59	42	3809.77

Supplementary Table 15. Geometry and vibrational frequencies of the transition state of THF·2H_2O^+ without solvent effect obtained by B3LYP/cc-pVDZ.

Geometry	Coordinates (Å)				Vibrational mode, Frequency(cm^{-1})			
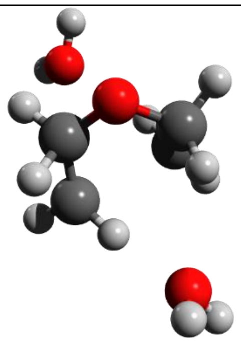	O	-0.16212000	0.52681100	1.88034700	1	-842.40	27	1079.42
	C	-0.66488000	-0.65393700	1.47816100	2	39.27	28	1127.79
	C	-0.34296200	-1.19575700	0.20763600	3	58.84	29	1194.33
	C	-0.51883900	1.26830700	-0.39757600	4	98.14	30	1219.14
	C	0.35461700	1.35932100	0.79796200	5	119.17	31	1251.81
	H	0.05966800	-1.62694300	1.66742800	6	126.52	32	1316.12
	H	-1.61634900	-0.91840500	1.95528500	7	166.31	33	1358.85
	H	0.62286200	-0.99161400	-0.28139300	8	175.73	34	1417.33
B3LYP/cc-pVDZ	H	-0.95264600	-2.02933000	-0.15940700	9	185.64	35	1444.97
	H	-0.10020100	1.45084300	-1.39020400	10	238.29	36	1464.40
	H	-1.60647000	1.24322600	-0.26167500	11	251.06	37	1531.68
	H	1.39766100	1.08867000	0.56947100	12	275.23	38	1659.03
	H	0.33786100	2.37910200	1.22222900	13	276.55	39	1659.30
	O	2.47712300	-0.52552700	-0.84846400	14	293.29	40	2200.86
	H	3.28222300	-0.89327200	-0.45007800	15	309.06	41	3035.02
	H	2.74189200	-0.33930400	-1.76341200	16	326.83	42	3054.30
	O	-3.34143100	0.49421800	1.03036500	17	435.41	43	3098.81
	H	-3.63311500	1.16645400	1.66714300	18	468.94	44	3118.72
	H	-4.16942400	0.21494000	0.60826300	19	636.37	45	3154.73
					20	746.80	46	3216.96
					21	765.24	47	3255.59
					22	841.44	48	3744.82
					23	917.28	49	3748.56
					24	970.14	50	3835.66
					25	1029.00	51	3837.40
					26	1048.94		

Supplementary Table 16. Geometry and vibrational frequencies of the transition state of $THF\cdot 2H_2O^+$ with solvent effect obtained by B3LYP/cc-pVDZ.

Geometry	Coordinates (Å)				Vibrational mode, Frequency(cm^{-1})			
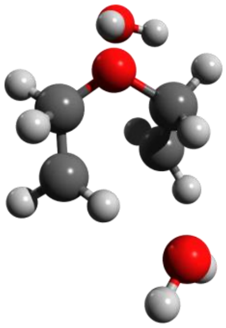	O	-0.69070000	0.32892100	1.78094500	1	-745.64	27	1087.98
	C	-0.71400600	-0.98920100	1.42243900	2	42.45	28	1121.65
	C	-0.01737900	-1.45751300	0.29064900	3	50.90	29	1186.96
	C	-0.69420100	0.84251500	-0.58898900	4	93.51	30	1199.72
	C	-0.15848900	1.21178400	0.74229100	5	107.54	31	1241.66
	H	0.19626500	-1.65116700	1.86252900	6	125.37	32	1311.21
	H	-1.60776800	-1.52026500	1.77128700	7	141.14	33	1352.17
	H	0.90836500	-0.97964900	-0.06510800	8	148.51	34	1404.11
B3LYP/cc-pVDZ	H	-0.26015400	-2.46356900	-0.06803200	9	186.67	35	1419.19
with solvent effect	H	-0.09453200	1.01134000	-1.48615900	10	202.27	36	1439.32
	H	-1.76287700	0.63260400	-0.68569300	11	206.27	37	1519.73
	H	0.94086000	1.19705900	0.76829300	12	227.92	38	1613.04
	H	-0.50927300	2.21278800	1.04630300	13	273.93	39	1650.22
	O	2.66062400	-0.00436500	-0.60218100	14	293.75	40	2345.83
	H	3.15736300	-0.78566500	-0.89748400	15	309.34	41	3050.31
	H	2.24023900	0.30513900	-1.42212200	16	384.23	42	3056.63
	O	-3.51294600	1.20381200	1.21879000	17	459.10	43	3120.40
	H	-2.70728800	0.91943100	1.68401200	18	461.78	44	3148.07
	H	-3.20863100	2.00380400	0.76031300	19	632.15	45	3162.63
					20	719.75	46	3232.94
					21	747.50	47	3275.52
					22	829.26	48	3708.45
					23	891.29	49	3728.97
					24	955.71	50	3796.01
					25	1031.14	51	3809.27
					26	1048.53		

Supplementary Table 17. Geometry and vibrational frequencies of the transition state of THF·3H_2O^+ without solvent effect obtained by B3LYP/cc-pVDZ.

Geometry	Coordinates (Å)				Vibrational mode, Frequency(cm^{-1})			
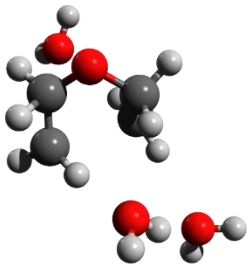	O	-0.16724600	0.45686100	1.93002000	1	-836.05	31	1037.14
	C	-0.63992500	-0.71870500	1.47792400	2	40.42	32	1057.04
	C	-0.23186500	-1.24845900	0.22680800	3	47.09	33	1101.07
	C	-0.29393400	1.22450700	-0.36844100	4	66.70	34	1136.62
	C	0.46689000	1.28112400	0.90499100	5	78.62	35	1195.43
	H	0.06769800	-1.69253900	1.71097000	6	109.74	36	1219.95
	H	-1.62402600	-0.98248900	1.88321600	7	124.71	37	1253.29
B3LYP/cc-pVDZ	H	0.76607900	-1.04362600	-0.20432000	8	138.24	38	1319.00
	H	-0.82376600	-2.07532200	-0.18277200	9	179.78	39	1364.23
	H	0.23280800	1.36894600	-1.31687200	10	197.79	40	1419.82
	H	-1.38908300	1.23998900	-0.32943700	11	202.54	41	1449.12
	H	1.51599100	0.97563400	0.76682400	12	217.62	42	1467.15
	H	0.44148500	2.29709900	1.33734300	13	233.83	43	1533.76
	O	2.49671800	-0.50419900	-0.77872800	14	241.72	44	1633.50
	H	3.35594700	-0.94973400	-0.75523000	15	246.39	45	1657.64
	H	2.50784200	0.04726800	-1.59185000	16	264.13	46	1670.13
	O	2.04773300	1.23351200	-2.88246500	17	291.19	47	2207.41
	H	1.87164100	0.92778300	-3.78672300	18	297.05	48	2987.92
	H	2.57572200	2.03890500	-3.00449400	19	315.66	49	3035.41
	O	-3.24939000	0.50674200	0.86637500	20	323.15	50	3099.63
	H	-3.53668600	1.17817300	1.50563200	21	465.12	51	3111.62
	H	-4.07163300	0.26911000	0.40954200	22	481.68	52	3155.83
					23	509.42	53	3204.27
					24	642.99	54	3245.27
					25	741.20	55	3550.25
					26	761.76	56	3746.78
					27	776.13	57	3748.01
					28	850.22	58	3825.52
					29	924.06	59	3838.95
					30	972.39	60	3839.78

Supplementary Table 18. Geometry and vibrational frequencies of the transition state of THF·3H_2O^+ with solvent effect obtained by B3LYP/cc-pVDZ.

Geometry	Coordinates (Å)				Vibrational mode, Frequency(cm^{-1})			
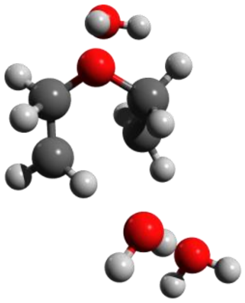	O	-0.62100700	0.20712500	1.77432800	1	-769.37	31	1039.54
	C	-0.67460800	-1.05326000	1.25246500	2	27.48	32	1057.06
	C	0.02249200	-1.39851800	0.07756100	3	48.60	33	1104.35
	C	-0.56852200	1.01162500	-0.51700100	4	57.29	34	1130.19
	C	-0.05098300	1.19983200	0.85909500	5	60.34	35	1187.37
	H	0.21815900	-1.79124200	1.60732300	6	83.27	36	1198.67
	H	-1.58406500	-1.60017800	1.52850300	7	113.55	37	1247.40
	H	0.97309500	-0.91595800	-0.20673000	8	130.81	38	1313.05
B3LYP/cc-pVDZ	H	-0.25264600	-2.34414000	-0.40256500	9	133.95	39	1354.17
with solvent effect	H	0.06649700	1.23427300	-1.37761100	10	165.28	40	1406.80
	H	-1.64328200	0.86156700	-0.65114900	11	185.40	41	1424.27
	H	1.04673900	1.15261600	0.90347200	12	191.78	42	1440.91
	H	-0.38625400	2.16289900	1.28007600	13	209.16	43	1524.98
	O	2.79219600	-0.02441600	-0.49863900	14	227.56	44	1607.51
	H	3.29397500	-0.79751100	-0.80392100	15	249.83	45	1633.01
	H	2.52310300	0.41734300	-1.34004100	16	263.53	46	1651.62
	O	1.90689900	1.21973500	-2.81629600	17	274.87	47	2326.34
	H	2.04439300	0.55697600	-3.51434200	18	298.47	48	3020.95
	H	2.54238100	1.91815200	-3.04815900	19	313.61	49	3051.89
	O	-3.46262100	1.07476300	1.17642600	20	425.55	50	3122.01
	H	-2.67638800	0.78561400	1.67078300	21	434.06	51	3138.89
	H	-3.19055300	1.95328300	0.86473400	22	444.22	52	3161.70
					23	465.81	53	3224.50
					24	642.07	54	3270.76
					25	679.32	55	3420.14
					26	732.83	56	3713.46
					27	761.00	57	3728.70
					28	832.65	58	3775.82
					29	900.02	59	3797.40
					30	964.03	60	3809.34

Supplementary Table 19. Geometry and vibrational frequencies of the transition state of THF·4H_2O^+ without solvent effect obtained by B3LYP/cc-pVDZ.

Geometry	Coordinates (Å)				Vibrational mode, Frequency(cm^{-1})			
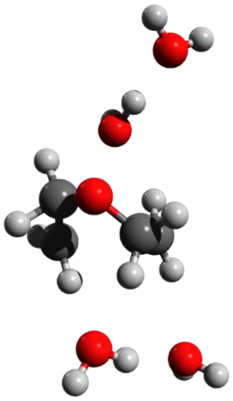	O	-0.18033600	0.68029900	1.73553400	1	-860.78	36	975.07
	C	-0.72576500	-0.48404300	1.34459800	2	13.18	37	1036.24
	C	-0.26768000	-1.15593200	0.18300900	3	25.13	38	1056.37
	C	-0.09417100	1.22579500	-0.62978000	4	44.33	39	1097.01
	C	0.57306600	1.36735200	0.68894800	5	51.92	40	1140.29
	H	-0.12400700	-1.49649600	1.70392400	6	72.44	41	1187.20
	H	-1.75952000	-0.62647400	1.67991600	7	78.84	42	1219.89
	H	0.77566000	-1.07413400	-0.17207200	8	97.81	43	1253.74
	H	-0.90026000	-1.95933900	-0.21120000	9	115.33	44	1319.31
	H	0.51226700	1.25234000	-1.54032900	10	134.18	45	1364.36
B3LYP/cc-pVDZ	H	-1.18871700	1.28777600	-0.67628700	11	142.10	46	1419.66
	H	1.60972400	0.99634000	0.66485600	12	179.91	47	1451.67
	H	0.57300200	2.42061000	1.02095100	13	189.17	48	1466.23
	O	2.59499500	-0.71577200	-0.64270300	14	198.31	49	1534.91
	H	3.41530700	-1.21190300	-0.50851600	15	211.14	50	1633.26
	H	2.70836100	-0.24723300	-1.49846600	16	220.98	51	1647.46
	O	2.44966500	0.83256700	-2.93367800	17	243.50	52	1669.65
	H	2.31693500	0.45160400	-3.81635100	18	249.41	53	1684.18
	H	3.04485600	1.58358400	-3.08760200	19	260.01	54	2183.32
	O	-3.10877800	0.76089400	0.32575700	20	264.04	55	3001.14
	H	-3.41336400	1.56926900	0.79972300	21	277.23	56	3036.02
	H	-3.88842500	0.49151200	-0.18230500	22	293.12	57	3096.00
	O	-3.95932100	3.05379400	1.61027800	23	305.91	58	3101.27
	H	-4.39254500	3.78250000	1.14066400	24	327.10	59	3160.37
	H	-4.39732700	3.03501000	2.47476200	25	432.74	60	3208.51
					26	467.11	61	3238.24
					27	478.26	62	3511.37
					28	515.19	63	3556.57
					29	644.64	64	3749.63
					30	737.71	65	3762.40
					31	758.72	66	3811.03
					32	772.72	67	3827.16
					33	803.75	68	3842.23
					34	850.65	69	3857.35
					35	926.63		

Supplementary Table 20. Geometry and vibrational frequencies of the transition state of THF·4H_2O^+ with solvent effect obtained by B3LYP/cc-pVDZ.

Geometry	Coordinates (Å)				Vibrational mode, Frequency(cm^{-1})			
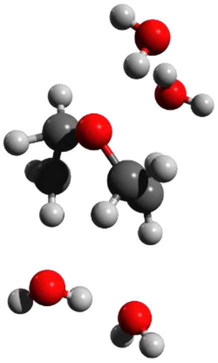	O	-0.51527000	1.19393400	1.60379400	1	-799.10	36	964.64
	C	-0.79266400	-0.13674900	1.48959600	2	28.72	37	1035.72
	C	-0.15909900	-0.93944500	0.52427100	3	32.08	38	1045.22
	C	-0.41288500	1.23032100	-0.82488000	4	44.28	39	1106.28
	C	0.16393400	1.76153400	0.43171700	5	55.79	40	1133.42
	H	-0.03374800	-0.86828400	2.09368600	6	58.48	41	1188.72
	H	-1.78223300	-0.41197900	1.87347300	7	71.00	42	1191.19
	H	0.85280900	-0.71541300	0.14529700	8	85.36	43	1238.53
	H	-0.58146500	-1.93469900	0.34850800	9	126.35	44	1309.87
B3LYP/cc-pVDZ	H	0.22275500	1.11306600	-1.70543700	10	133.90	45	1346.83
with solvent effect	H	-1.50125700	1.13693100	-0.90952200	11	148.43	46	1408.06
	H	1.24503900	1.57279500	0.51234700	12	168.81	47	1431.79
	H	-0.01683800	2.84570300	0.52935000	13	203.19	48	1444.69
	O	2.77909900	-0.25962200	-0.37709300	14	216.30	49	1524.97
	H	3.11732800	-1.16027600	-0.50888900	15	220.21	50	1611.02
	H	2.54696300	0.03123200	-1.29212600	16	233.41	51	1639.69
	O	2.12634500	0.55789700	-2.94961000	17	236.64	52	1640.20
	H	2.37959200	-0.17358600	-3.53791100	18	237.03	53	1648.15
	H	2.77401000	1.24935800	-3.16906400	19	243.98	54	2305.54
	O	-3.79622700	0.97650200	-0.52295300	20	271.44	55	3021.51
	H	-3.62160200	1.39369400	0.35441900	21	283.72	56	3050.37
	H	-3.76608500	1.73745900	-1.12518200	22	296.05	57	3114.77
	O	-3.24651500	2.13482400	1.95372200	23	326.79	58	3119.17
	H	-3.53197800	1.46281500	2.59574600	24	442.97	59	3163.47
	H	-2.27638800	2.02190700	1.94036900	25	455.47	60	3228.22
					26	462.55	61	3257.64
					27	473.01	62	3423.22
					28	518.80	63	3426.76
					29	643.58	64	3652.83
					30	682.00	65	3727.64
					31	729.91	66	3775.02
					32	746.93	67	3775.75
					33	757.46	68	3777.00
					34	831.75	69	3807.30
					35	907.24		

Supplementary Note 6. Experimental branching ratios

We compare the fragmentation pattern of hydrated THF and pure THF clusters to that of isolated THF in the mass range from 24 u to 50 u. The intact $C_4H_8O^+$ ion yields are normalized to 1, the total fragment ion yields from 24 u to 50 u are about 6.82 for isolated THF [3], 6.98 for pure THF clusters, and 7.43 for hydrated THF. This means that the fragmentation patterns in the mass range from 24 to 50 u are most likely due to the dissociation of isolated THF. While slightly increased intensity in hydrated THF can be partly ascribed to the water clusters ion fragments like $(H_2O)_2H^+$ at m/z 37.

Supplementary Table 21. Branching ratios of fragment ions relative to THF^+.

Isolated THF		Pure THF		Hydrated THF	
Mass	intensity	mass	intensity	mass	intensity
24-50	6.82	24-50	6.98	24-50	7.43
				62	0.036
71	0.95	71	1.20	71	1.24
72	1.00	72	1.00	72	1.00
		73	0.74	73	0.25
				91	0.064
				109	0.015
		116	0.27	116	0.045
				127	0.0046
		145	0.53	145	0.047

Supplementary Note 7. Ionization and fragmentation of ethanol

In order to demonstrate that similar effects as observed for THF dimers also show up in other hydrogen-bonded molecular dimers we consider exemplarily the ethanol dimer. By using the same experimental methods, we performed experiments of ionization and fragmentation of ethanol monomer and dimers induced by electron-impact. It can be seen from the measured binding energy (BE) spectra that the HOMO ionization of ethanol (BE ~ 10.7 eV) leads to the intact $C_2H_6O^+$, in Supplementary Figure 6c. The C-C bond breaking channel in the isolated ethanol molecule requires significantly more energy. Thus, the BE for CH_3O^+ production is about 13.5 eV as seen in Supplementary Figure 6b corresponding to the ionization of the HOMO-2 and HOMO-3 orbitals. The same C-C bond-breaking channel in the ethanol dimer which gives rise to the $(C_2H_6O){\cdot}CH_3O^+$ ion fragment shows significantly lower BE with a peak located at about 10.7 eV. These results reveal a similar effect as observed in our study on THF.

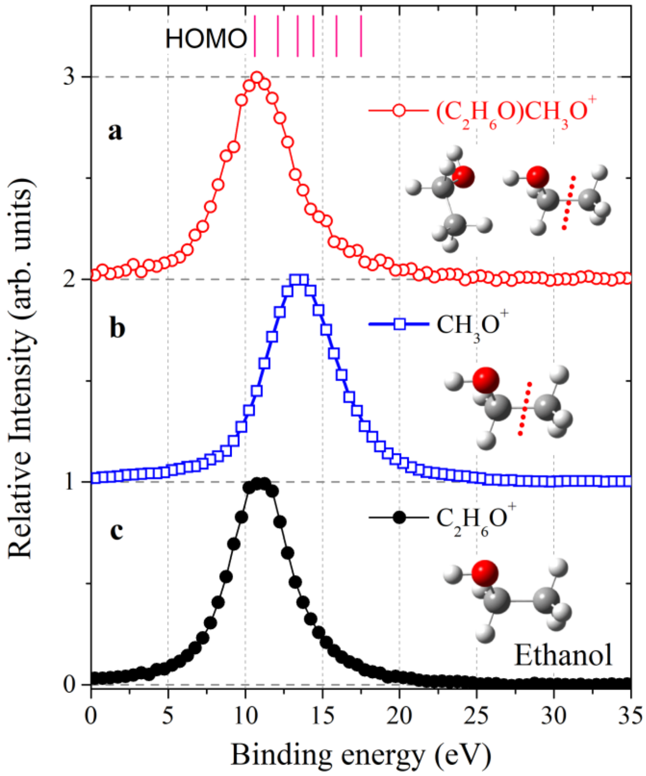

Supplementary Figure 6. Measured binding energy spectra for various fragment species upon ionization of ethanol. **a** corresponds to ionization in an ethanol dimer and subsequent dissociation to $(C_2H_6O){\cdot}CH_3O^+$, i.e. the C-C bond breaking channel. **b** and **c** are ionization of ethanol monomers and subsequent dissociation to CH_3O^+ and non-dissociated $C_2H_6O^+$, respectively. The vertical lines at the top of the figure are the valence orbital ionization energies of the ethanol monomer. The HOMO refers to the highest occupied molecular orbital. Error bars are defined as s.d. and mostly smaller than the symbol size.

Supplementary References

[1] M. Barbatti, et al, WIREs: Comp. Mol. Sci. 4, 26 (2014).
[2] M. Barbatti, et al, J. Photochem. Photobio. A 190, 288 (2007).
[3] M. Dampc, et al., J. Phys. B: At. Mol. Opt. Phys. 44, 055206 (2011).
